# Mechanisms of Beta Cell Dysfunction Associated With Viral Infection

**DOI:** 10.1007/s11892-015-0654-x

**Published:** 2015-08-18

**Authors:** Antje Petzold, Michele Solimena, Klaus-Peter Knoch

**Affiliations:** Paul Langerhans Institute Dresden of the Helmholtz Center Munich at University Hospital Carl Gustav Carus and Faculty of Medicine, Technische Universität Dresden, Fetscherstr.74, 01307 Dresden, Germany; German Center for Diabetes Research (DZD e.V.), Neuherberg, Germany; Max Planck Institute of Molecular Cell Biology and Genetics, Dresden, Germany

**Keywords:** Type 1 diabetes, Beta cell, Enterovirus, Coxsackievirus

## Abstract

Type 1 diabetes (T1D) results from genetic predisposition and environmental factors leading to the autoimmune destruction of pancreatic beta cells. Recently, a rapid increase in the incidence of childhood T1D has been observed worldwide; this is too fast to be explained by genetic factors alone, pointing to the spreading of environmental factors linked to the disease. Enteroviruses (EVs) are perhaps the most investigated environmental agents in relationship to the pathogenesis of T1D. While several studies point to the likelihood of such correlation, epidemiological evidence in its support is inconclusive or in some instances even against it. Hence, it is still unknown if and how EVs are involved in the development of T1D. Here we review recent findings concerning the biology of EV in beta cells and the potential implications of this knowledge for the understanding of beta cell dysfunction and autoimmune destruction in T1D.

## Introduction

In type 1 diabetes (T1D), the insulin-producing pancreatic beta cells are destroyed by misguided immune cells. The incidence of childhood T1D has increased annually by ∼3 %, worldwide, although regional differences exist [1, 2]. Genetic as well as environmental factors affecting the immune system and possibly beta cells contribute to the development of the disease. More than 40 genetic loci associated with T1D have been identified including those of insulin, cytotoxic T lymphocyte antigen 4 (CTLA-4), interleukin 2 receptor a (IL2RA), tyrosine phosphatase PTPN22, and the viral double-stranded RNA (dsRNA) sensor IFIH1 [3]. However, human leukocyte antigen (HLA) loci, and in particular selected HLA class II DR and DQ alleles, confer the strongest risk being associated with ∼40 % of T1D cases, clearly indicating that susceptibility for the disease is at least partially inherited [4]. Furthermore, regional differences in the incidence of T1D can be attributed, at least in part, to the varying frequencies of HLA haplotypes among different populations [5]. On the other hand, studies conducted in several countries have revealed that concordance for the disease among monozygotic twins does not exceed 50 %, as it would be expected if T1D were to be caused by genetic factors alone [6, 7]. Additionally, migration studies have shown that children of immigrants, who moved from an area with a low incidence to an area of high incidence of T1D, increased their risk for developing the disease compared to children in the area of origin [8, 9]. Taken together, these data point to the contribution of environmental factors toward the onset and/or progression of autoimmunity directed against beta cells.

### Viruses as Agents for T1D

Several viruses, including cytomegalovirus [10], Epstein-Barr virus [11], mumps virus [12, 13], rotavirus [14], and rubella virus [15] are among the environmental factors thought to foster the development of T1D. Above all, enteroviruses (EVs), and especially coxsackievirus B (CVB), have been linked to the disease.

Animal models for virus induction of diabetes exist since 1968, when it was first shown that infection of adult mice with encephalomyocarditis virus resulted in persistent hyperglycemia secondary to degranulation and focal necrosis of islet beta cells [16]. Subsequent studies in the non-obese diabetic (NOD) mouse, which spontaneously develops insulitis and autoimmune diabetes, pointed to the ability of both CVB3 and CVB4, also picornaviruses, to accelerate the onset of T1D in old prediabetic animals with established insulitis [17, 18]. On the other hand, infection of young NOD mice with different strains of CVB3 and CVB4 reduced the incidence of T1D onset [19]. From these data, one can conclude that the timing of infection might play a major role. Nevertheless, also various other factors such as the viral dose, the viral strain, and/or the species infected by the virus might be of importance. Findings from animal models might not necessarily reflect the conditions in human and, therefore, extrapolation from one species to another should be evaluated carefully.

In 1969, Gamble and colleagues first reported the occurrence of higher titer of neutralizing antibodies against CVB4 in recently diagnosed T1D patients [20]. These authors also noted a seasonal pattern of T1D onset with incidence increasing in late fall and early winter following outbreaks of CVB infection [21]. A decade later, Yoon and colleagues isolated CVB4 from the pancreas of a child, who died from diabetic ketoacidosis immediately after the onset of the disease, and induced diabetes in mice infected with the isolated virus [22]. This was followed by further serological studies showing a correlation between T1D and EV infection [23, 24]. However, a meta-analysis including 26 serological case-control studies found no convincing evidence for a correlation between CVB serology and T1D [25]. Yet, blood samples from recent-onset T1D patients were found positive for enteroviral RNA by PCR [26, 27]. Moreover, enteroviral RNA and capsid protein VP1 were detected in islets of pancreatic autopsy specimens from patients with T1D [28–30]. In addition, another meta-analysis of 24 retrospective and prospective studies found a significant association between T1D-related autoimmunity and EV infection, as detected by measuring enteroviral RNA or protein in stool, blood, or tissue [31]. Conversely, next-generation sequencing of plasma samples from children with rapid-onset T1D did not provide evidence for correlation with enteroviral infection [32]. More recently, Krogvold et al. reported the expression of VP1 in <2 % of islets and low levels of enteroviral RNA in pancreatic biopsies from seven T1D subjects and thus postulated that a low-grade infection of islet cells contributes to the development of the disease [33••]. On the other hand, EV infection might be responsible for a fulminant form of T1D reported in Japan, which is characterized by massive beta cell destruction in the absence of autoantibodies against beta cell antigens [34, 35].

As in mice, some CVB strains may protect from the development of T1D in humans. In Finnish children, the presence of neutralizing antibodies against CVB1 was recently shown to be associated with an increased risk of beta cell autoimmunity, while neutralizing antibodies to CVB3 and CVB6 correlated with a reduced risk for T1D [36••]. Due to the close phylogenetic relatedness of these three CVB serotypes, the authors suggested that CVB3/CVB6-specific T cells may induce an immunological cross-protection against the diabetogenic effect of a later CVB1 infection. Similar findings were also reported in a second study, which found the frequency of antibodies against CVB1 to be higher in diabetic children compared to controls [37•].

These discrepancies about the role of EVs in the development of T1D in humans could reflect the different effects of different strains in different populations and the limitations of many studies (size of cohorts, types, timing, and frequency of sampling). Most recently, a nationwide, population-based cohort study in Taiwan concluded that EV infection is associated with an increased risk of childhood T1D [38]. Also, repeated infections rather than a single event as well as the concomitance of other predisposing environmental factors might be needed for the development of the disease, which would make more difficult to prove an association. Furthermore, laboratory protocols have not been standardized and thus thresholds for detection may vary considerably, while transient infections may escape detection. Hence, despite the progressive applications of more sensitive and accurate methodologies, a consensus about a link between viral infection and the onset of T1D has yet to be reached. In light of this uncertainty, the acquisition of knowledge about the biology of viruses in beta cells may be useful.

## Enteroviruses

The genus of *Enterovirus* belongs to the family of *Picornaviridae* and is grouped into 12 species named *Enterovirus A-J* and *Rhinovirus A-C*, for a total of >100 serotypes including poliovirus, echovirus, coxsackievirus A and B, and others [39]. EVs are common in humans and infect billions of people every year. Among them, CVB has been the most frequently associated with T1D. CVB can cause acute inflammatory diseases like myocarditis, meningitis, and pancreatitis but mostly induce milder symptoms such as fever, summer cold, or rash, or is completely asymptomatic [40, 41]. They are transmitted mainly via the fecal-oral route and replicate primarily in the intestine and secondary target organs like the pancreas [22].

CVB seems to exhibit a specific tropism for the pancreas and beta cells in particular. For example, CVB4 is able to infect and replicate in human pancreatic islets in vitro [42]. As mentioned above, CVB3 RNA was detected in islet of autopsy pancreata from T1D patients and children who died from fulminant CV infection, but not in exocrine tissue [28]. Recently, this was corroborated by evidence of CVB5 replication exclusively in human endocrine islets, but not in exocrine clusters [43]. Additionally, VP1 was observed in beta but not alpha cells of islets from recent-onset T1D patients [29, 30]. A possible explanation for this observation is the ability of alpha cells to mount a more efficient antiviral response to CVB4 and B5 than beta cells and thus be better able to clear viral infections [44•].

**Fig. 1:**
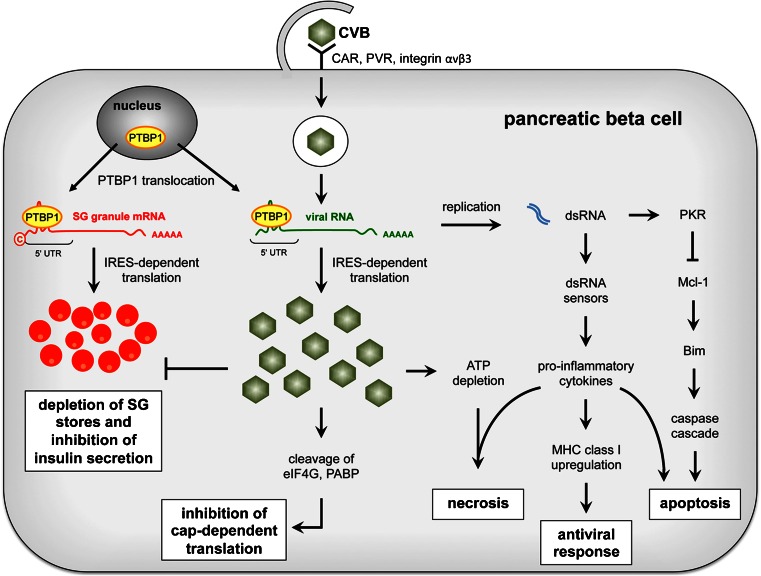
Impact of CVB infection on beta cell function and survival. CVB binds to CAR, PVR, and/or integrin αvβ3 at the plasma membrane of the beta cell. Upon entry and translocation into the cytoplasm, the sense-stranded CVB RNA is translated in a PTBP1-mediated, IRES-dependent fashion by the host machinery. Cap-dependent translation of host cell proteins is inhibited through cleavage of eIF4G and PABP. Glucose-stimulated translation of insulin secretory granule (SG) cargoes is, however, unaffected being itself cap-independent and reliant on PTBP1, which in CVB-infected cells undergoes a massive nucleo-cytoplasmic translocation. Insulin secretion is nevertheless impaired and granule stores are depleted due to the targeting of their cargo proteins to intracellular disposal. Recognition of viral dsRNA by dsRNA sensors activates the antiviral response with production and secretion of pro-inflammatory cytokines and upregulation of MHC class I molecules. Additionally, dsRNA activates PKR with inhibition of the antiapoptotic Mcl-1 and thereby release of pro-apoptotic Bim and activation of the mitochondrial caspase cascade leading to apoptosis. Apoptosis can be further induced by pro-inflammatory cytokines and switch to necrosis in case of concomitant ATP depletion

CVBs are small, non-enveloped, positive-stranded RNA viruses containing an icosahedral capsid of ∼30 nm in diameter that consist of four viral proteins (VP1-VP4) [40]. To invade the host cell, they primarily use the coxsackievirus and adenovirus receptor (CAR) [45] but also the decay-accelerating factor (DAF) [46] (Table 1). CAR is expressed in both alpha and beta cells of human pancreatic islets [47] while DAF has not been detected in human islets [28]. Poliovirus receptor (PVR) and integrin αvβ3 can also mediate CVB entry, as antibodies against them protected human beta cells from CVB4 and CVB5 infection [28] (Fig. 1).

**Table 1:** Interactions of enteroviral components with host cellular structures including references specific for beta cells

Viral component	Function	Affected cellular structure	Result	References	Ref. for beta cells
VP1–VP4	Capsid proteins	CAR, DAF, PVR, integrin αvβ3	Cell entry of the virus	[28, 45–47]	[28, 47]
VP2	Capsid protein	Siva	Activation of apoptosis	[94]	
2A	Protease	eIF4GI, eIF4GII, PABP	Inhibition of cap-dependent host translation	[53, 56, 95]	[63••]
		cytokeratin 8	Changes in cytoskeletal network	[96]	
		MDA5, MAVS	Inhibition of type I IFN response of the host	[97, 98]	
		NUP63, NUP98, NUP153	Disruption of nucleo-cytoplasmic RNA transport	[99, 100]	
		PARP, caspase-3	Activation of apoptosis	[101]	
		SQSTM1/p62	Inhibition of selective autophagy	[102]	
2B/2BC	Enhancer of cell	COPI, COPII	Formation of viral vesicles at the ER	[103, 104]	
	permeability	ER/Golgi membranes	Pore formation, Release of Ca^2+^ from ER and Golgi	[105–107]	
3A	Inhibitor of intracellular protein transport	Arf, GBF1, COPI	Inhibition of protein secretion and MHC class I antigen presentation	[108–111]	
3C	Protease	eIF4A1, eIF5B, PABP	Inhibition of cap-dependent host translation	[54, 112, 113]	
		MAP-4	Depolarization of microtubule network	[114]	
		RIG-I, MAVS, TRIF, TAB2 complex, TRIF	Inhibition of type I IFN response of the host	[98, 115–117]	
		RNA-binding proteins e.g. PTB, PCBP	Inhibition of IRES-mediated translation to switch to viral replication	[118, 119]	
		Transcription factors e.g. TFIIIC, TBP	Shut-off of host cell transcription	[120–122]	
		PARP, caspase-3	Activation of apoptosis	[101, 123]	
3D	RNA polymerase	Sam68	Induction of PI3K/Akt signaling	[124]	
3CD	Protease, RNA	RNA-binding proteins, e.g., PCBP2 and AUF1	Switch from translation to replication	[125, 126]	
	polymerase	Arf, BIG1, BIG2	Reorganization of membranes for viral replication	[108, 110, 127]	
ssRNA	viral genome	RNA-binding proteins e.g. PTB, La, UNR, PCBP2	Initiation of IRES-mediated translation	[50, 51, 63••]	[63••]
dsRNA	replication intermediate	PKR	Activation of apoptosis	[60, 61•]	[60, 61•]

Once the virus has adsorbed to the cell surface, its capsid proteins undergo conformational changes that enable its RNA genome to enter the cell, presumably through the formation of a pore within the host plasma membrane. Additionally, intake of CVB by a combination of endocytosis and macropinocytosis at tight junctions or lipid rafts has been discussed [40] [48, 49]. However, the detailed process of entry remains to be clarified. Upon genome translocation into the host cell, the sense-strand viral RNA is translated and thereafter replicated in the cytosol by exploiting the protein machinery of the host due to the limited coding capacity of its own genome. Being uncapped and lacking a poly-A tail, viral RNAs are translated in a cap-independent fashion [50] (Fig. 1). Their 5′-untranslated region, in particular, contains internal ribosome entry sites (IRESs) for the binding of IRES-transacting factors (ITAFs), such as polypyrimidine tract-binding protein 1 (PTBP1, also referred to as PTB), which promote the recruitment of ribosomes to the viral RNA for translation [51]. The viral genome encodes a single polypeptide that is cleaved into several proteins by self-activated viral proteases 2A and 3C [41]. Proteases 2A and 3C, in turn, shut down the cap-dependent translation of host mRNAs by cleaving eukaryotic translation initiation factors (eIFs) eIF4GI [52, 53] and eIF5B [54] as well as the poly(A)-binding protein (PABP) [55, 56] (Table 1). In this way, EVs can exploit the host translation machinery to their advantage.

Next, the four structural viral proteins VP1–VP4 self-assemble into empty capsids, whereas non-structural proteins mediate the transcription of the positive-strand RNA genome. Through RNA encapsidation and conformational changes, stable, infectious virions are generated [41]. The mechanism of viral particle release from infected cells is still unknown, and several possibilities have been postulated including changes in cell membrane permeability, lysis, and apoptosis [57, 58].

## Dysfunction of EV-Infected Beta Cells

### Beta Cell Death and Proliferation

EVs impair beta cell function and display different cytolytic effects in pancreatic beta cells with some serotypes being highly cytolytic while others replicating without apparently destroying the cell [59]. Infection of beta cells with CVB5 induces cell death via activation of the viral sensor protein kinase R (PKR), thereby reducing the expression of the antiapoptotic protein myeloid leukemia cell sequence 1 (Mcl-1) [60] (Fig. 1). Concomitantly, the pro-apoptotic protein Bim is released and activates the caspase cascade of the mitochondrial apoptosis pathway (Table 1). This was confirmed in VP1-positive beta cells of pancreatic islets from T1D patients, in which PKR was upregulated and Mcl-1 depleted [61•]. Alternatively, productive infection with CVB3, CVB4, and CVB5 can induce beta cell death in human islets with morphological changes characteristic for pyknosis, in the absence of signs for apoptosis [62]. Likewise, murine insulinoma cells infected with CVB5 died from necrosis, but not apoptosis [63••]. Furthermore, gene expression profiling of human islets infected with a lytic EV strain revealed an upregulation of pro-inflammatory cytokines interleukin 1α (IL-1α), IL-1β, and tumor necrosis factor α (TNF-α) with coeval enhanced apoptosis and necrosis, the latter resulting from the depletion of ATP concomitantly with the otherwise pro-apoptotic action of the cytokines [64••] (Fig. 1).

The generation of double-stranded RNA (dsRNA) during the process of viral replication elicits an innate immunity response through the activation of dsRNA sensors, such as Toll-like receptor 3 (TLRs), retinoic-acid-inducible gene I (RIG-I), or melanoma differentiation associated protein 5 (MDA5, also referred to as IFIH1, Table 1), all of which are indeed upregulated in human pancreatic islets upon CVB5 infection [64••, 65, 66] (Fig. 1). Activation of dsRNA sensors induces the production and secretion of cytokines and thereby initiates inflammation. Accordingly, pro-inflammatory cytokines IL-1α, IL-1β, IL-6, IL-8, TNF-α, and type I interferons (IFNs) IFN-α and IFN-β have been detected in human islets following their infection with CVB3, CVB4, or CVB5 [47, 64••, 66–68]. Among other effects, type I IFNs upregulate the expression of major histocompatibility complex (MHC) class I molecules, thus favoring the presentation of viral antigens at the cell surface of infected beta cells and consequently their death by activated cells of the immune system, including T cells. Notably, several studies have documented the overexpression of MHC class I in islets of T1D patients [30, 33••, 69]. On the other hand, overexpression of MHC class I could also enhance the recognition of beta cell autoantigens by autoreactive CD8-positive cytotoxic T cells, which have been found in pancreatic islets of T1D patients [70].

The modality of viral-induced cell death depends also on the multiplicity of infection (MOI) [71]. High MOI of isolated beta cells and human islets with CVB5 resulted predominantly in necrosis and transient apoptosis. Conversely, a low MOI correlated with moderate necrosis while apoptosis increased with time. Hence, apoptosis may not play a major role during a productive infection but be more relevant in the case of viral persistence in the tissue.

Intriguingly, there are also studies suggesting that EV infection may stimulate beta cell proliferation. Specifically, beta cells positive for the proliferation marker Ki67 were observed in VP1-positive islets isolated from recent-onset T1D patients [72]. However, both markers were not found within the same cell. Therefore, it was suggested that factors associated with viral replication might stimulate the proliferation of neighboring, non-infected beta cells. Additionally, infection of SJL mice with a diabetogenic or with a non-diabetogenic CVB4 strain caused the acute destruction of exocrine pancreas, while islets were largely spared [73]. In the longer term, however, the infection with the non-diabetogenic strain was associated with islet neogenesis, while in the case of the diabetogenic strain, islets were also destroyed. Based on these observations, lack of beta cell neogenesis upon viral infection could contribute to beta cell depletion in mice.

### Insulin Production and Secretion

Infection of isolated human islets with either CVB3, CVB4, or CVB5 reduced their insulin content and glucose-stimulated insulin secretion [42, 62]. CVB4-infected human islets transplanted in mice showed reduced insulin levels and hyperglycemia resulting in diabetes [74]. Accordingly, islets of T1D patients positive for CVB4 displayed also lower insulin release [29]. Gene expression profiling suggests that CVB infection downregulates factors involved in intracellular Ca^2+^ homeostasis and membrane potential as cause for impaired insulin secretion [64••]. In addition to insulin, other mature granule cargoes were depleted in CVB5-infected mouse islet and insulinoma cells, albeit glucose-stimulated translation of their precursor species, such as proinsulin, was unaffected [63••]. This is still possible because transcripts for most insulin granule cargoes, unlike those for most other beta cell proteins, but similar to the EV genome, are translated in a PTBP1-dependent, cap-independent fashion (Fig. 1). Massive relocation of PTBP1 from the nucleus to the cytosol of CVB-infected beta cells accounts for the availability of PTBP1 in amounts sufficient to support simultaneously the translation of both viral and granule cargo transcripts. The depletion of mature granule cargoes in infected cells indicated that CVB is nevertheless able to divert the traffic of these proteins along the secretory pathway and destine them to degradation prior to secretion, which is inhibited. In some other instances, however, CVB infection could also increase the levels of released insulin, possibly due to discharge of the hormone upon beta cell damage [42].

## Virus-Induced Pathogenesis in T1D

While we have acquired substantial knowledge on how EVs can directly exert a detrimental impact on beta cell function and viability, we still lack a mechanistic explanation for how this infection could also trigger or aggravate the loss of self-tolerance toward beta cells. To account for these scenarios, several hypotheses have been proposed.

According to one hypothesis, viral-induced damage of infected beta cells and inflammation enhance the presentation of released beta cell peptides by professional antigen-presenting cells [75]. Concomitantly, infected beta cells upregulate their expression of MHC class I molecules in order to facilitate the presentation of viral peptides, and thus their recognition and destruction by T cells and the clearance of the virus. This process, however, might also enhance the antigen presentation of peptides derived from secretory granule cargoes, which in infected cells are still being translated but also massively degraded [63••]. Whether and how such skewed presentation of granule-derived peptides accounts for the preferential loss of tolerance in T1D toward the granule cargoes proinsulin, insulin, IA-2/ICA512, IA-2β/phogrin, and Znt8/SLC30A8 remains to be investigated.

The related “bystander hypothesis” envisions that the release of pro-inflammatory cytokines and nitric oxide leading to insulitis and beta cell death could also follow the infection of neighboring pancreatic cells [76, 77]. In particular, there is evidence that enhanced release of islet autoantigens upon CVB4 infection stimulates pre-existing autoreactive T cells and accelerates the onset of T1D in NOD mice carrying an islet-autoantigen-specific T cell receptor transgene [78]. However, this mechanism has been challenged in view of the high number of pre-existing autoreactive T cells required to foster disease progression [17]. Additionally, cytokines secreted by infected cells or inflammatory cells are unlikely alone to break self-tolerance [79, 80].

An alternative mechanism known as “molecular mimicry” implies that loss of self-tolerance may occur due to short sequence similarities between viral proteins and endogenous proteins of beta cells. Potential T cell cross-reactivity has been documented between the P2-C protein of CVB4 and glutamic acid decarboxylase 65 (GAD65), a major autoantigen in T1D [81]. This hypothesis has been challenged due to experimental evidence indicating that neither autoantibodies nor autoreactive T cell clones isolated from T1D patients and specific for GAD65 epitopes cross-reacted with the CVB4 P2-C antigen [82, 83]. Molecular mimicry has also been proposed to occur between the rotavirus VP7 protein and IA-2/ICA512, an intrinsic protein of insulin granules and another major target of autoimmunity in T1D [84]. For instance, IA-2-restricted T cells were shown to proliferate upon exposure to a VP7 peptide and vice versa [85]. Additionally, IA-2 and its paralogue IA-2β/phogrin, also a T1D autoantigen, display sequence similarities with the enteroviral VP1 and VP0 precursor proteins with humoral cross-reactivities also occurring in both directions [86].

Prolonged inflammation due to viral persistence, replication, and antigenic stimulation has also been suggested as a potential mechanism leading to autoimmunity. For instance, some CVB strains have been reported to persist in human pancreatic islets [42, 47], presumably because of amino acid substitutions located at the surface of VP1 close to the predicted receptor binding canyon [87] or due to 5′ terminal deletions of the genome, which were shown to result in slower viral replication and loss of cytopathic effect [88]. Moreover, enteroviral RNA and VP1 have been detected in autopsy pancreata of T1D patients even beyond the stage of acute infection [28, 30]. EV can also persist in the intestine [89, 90] and blood cells [91]. These tissues may therefore represent chronic reservoirs from which EV propagates to other organs, such as the pancreas, leading overtime to beta cell autoimmunity. Loss of tolerance of autoreactive T cells against beta cell antigens may also result from deficits in central tolerance secondary to persistent viral infection of thymic cells. CVB4 has been shown to replicate and persist in fetal thymus organ cultures and thymic epithelial cells, thereby impairing T cell maturation and differentiation [92] and thus decreasing the production of insulin-like growth factor 2 (IGF-2), which might be involved in the establishment of central tolerance toward insulin [93].

## Conclusions

In recent years, increasing knowledge has been acquired on how viruses, and especially EVs, can infect beta cells and thus promote insulitis, impair insulin secretion, and trigger beta cell death. However, we still lack mechanistic insight into how a self-limiting acute viral infection, or even a latent virus-induced insulitis, may occasionally evolve into autoimmunity toward beta cells and thus cause T1D. So far, none of the hypotheses accounting for virus-induced beta cell autoimmunity has been supported by stringent evidence in humans, and the involvement of several mechanisms rather than just one is also plausible. Hence, further studies on the potential link between EV infections and T1D are warranted before strategies such as vaccines or antiviral drugs may be pursued as a means to prevent or halt the development of the disease.
